# UniProt: a worldwide hub of protein knowledge

**DOI:** 10.1093/nar/gky1049

**Published:** 2018-11-05

**Authors:** The UniProt Consortium

**Affiliations:** 1 European Molecular Biology Laboratory, European Bioinformatics Institute (EMBL-EBI), Wellcome Genome Campus, Hinxton, Cambridge CB10 1SD, UK; 2 SIB Swiss Institute of Bioinformatics, Centre Medical Universitaire, 1 rue Michel Servet, CH-1211 Geneva 4, Switzerland; 3 Protein Information Resource, Georgetown University Medical Center, 3300 Whitehaven Street NW, Suite 1200, Washington, DC 20007, USA; 4 Protein Information Resource, University of Delaware, 15 Innovation Way, Suite 205, Newark DE 19711, USA

## Abstract

The UniProt Knowledgebase is a collection of sequences and annotations for over 120 million proteins across all branches of life. Detailed annotations extracted from the literature by expert curators have been collected for over half a million of these proteins. These annotations are supplemented by annotations provided by rule based automated systems, and those imported from other resources. In this article we describe significant updates that we have made over the last 2 years to the resource. We have greatly expanded the number of Reference Proteomes that we provide and in particular we have focussed on improving the number of viral Reference Proteomes. The UniProt website has been augmented with new data visualizations for the subcellular localization of proteins as well as their structure and interactions. UniProt resources are available under a CC-BY (4.0) license via the web at https://www.uniprot.org/.

## INTRODUCTION

The proteins expressed in a cell at any moment of time determine its function, its topology, how it reacts to changes in environment and ultimately its longevity and well-being. Improvements in experimental techniques are providing ever deeper information on the structure and function of individual proteins, whilst large-scale sequencing efforts are driving increased coverage of the complete proteomes of the breadth of organisms that populate the tree of life. Our challenge is to capture the growing depth and breadth of information and make it easily available and interpretable to our users. The UniProt Knowledgebase (UniProtKB) combines reviewed UniProtKB/Swiss-Prot entries, to which data have been added by our expert biocuration team, with the unreviewed UniProtKB/TrEMBL entries which are annotated by automated systems including our rule-based systems. The output from large-scale sequencing projects form the vast majority of the ∼120 million entries in UniProtKB/TrEMBL. Improved metagenomic assembly and binning tools are resulting in an increasing number of high-quality metagenomic assembled genomes (MAGs) being represented in the database. Additionally, we provide the UniRef databases that cluster sequence sets at various levels of sequence identity and the UniProt Archive (UniParc) that delivers a complete set of known sequences, including historical obsolete sequence.

We describe the major developments that we have made since our last update published in this journal in 2017 (1) with a focus on how we are positioning the UniProt database to address the challenges of the increased volume of sequence data entering the database.

## PROGRESS AND DEVELOPMENTS

### Growth of sequences in UniProt

The UniProtKB Proteomes portal (https://www.uniprot.org/proteomes/) provides access to proteomes for over 84 thousand (84 387, release 2018_07) species with completely sequenced genomes. The majority of these proteomes are based on the translation of genome sequence submissions to the INSDC source databases—ENA, GenBank and the DDBJ (2). To ensure comprehensiveness, complementary pipelines have been developed to supplement these with genomes sequenced and/or annotated by groups such as Ensembl (3), Vectorbase (4) and WormBase ParaSite (5). This has been extended to RefSeq genomes, allowing us to import key genomes of special interest annotated by NCBI’s Eukaryotic Genome Annotation Pipeline. Release 2018_03, for example, saw the inclusion of a set of primate proteomes and a dozen genomes for marine mammals were imported from RefSeq for release 2018_08. Taxa may be specifically targeted by curators to fill gaps in the taxonomic space and additional proteome import can be requested by individual users.

**Figure 1.**
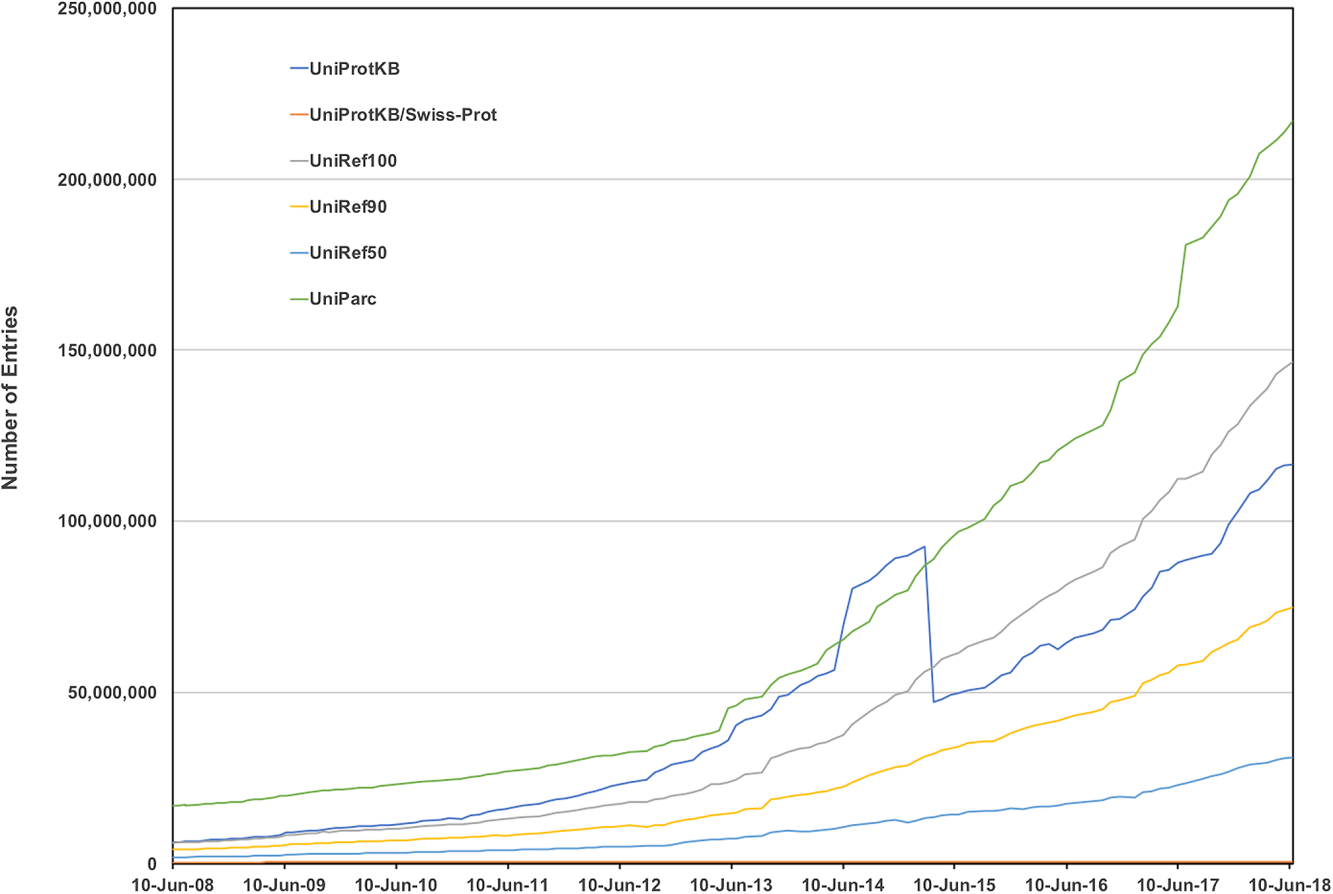
Growth of UniProt sequences over the last decade.

The continuing growth of sequenced genomes is a challenge for databases as much of this growth is being driven by sequencing of very similar and almost identical strains (90% of proteins have >90% identity) of the same species. We continue to see exponential growth in many of our datasets, see Figure 1. We are managing this growth by a number of processes. Most critically, a redundancy removal process was first introduced in 2015. This process identifies and removes almost identical proteomes of the same species before their inclusion in UniProtKB (https://www.uniprot.org/help/proteome_redundancy) and places their sequences in UniParc. Currently this process has removed ∼38% all complete proteomes (∼241 million proteins) from UniProtKB. As can be seen in Figure 1, the redundancy reduction both greatly reduced the size of UniProtKB as well as made its growth more scalable. This approach has now been extended from prokaryotes to fungi, resulting in the deprecation of ∼1 million fungal protein records in release 2016_08.

### Reference proteomes

**Figure 2.**
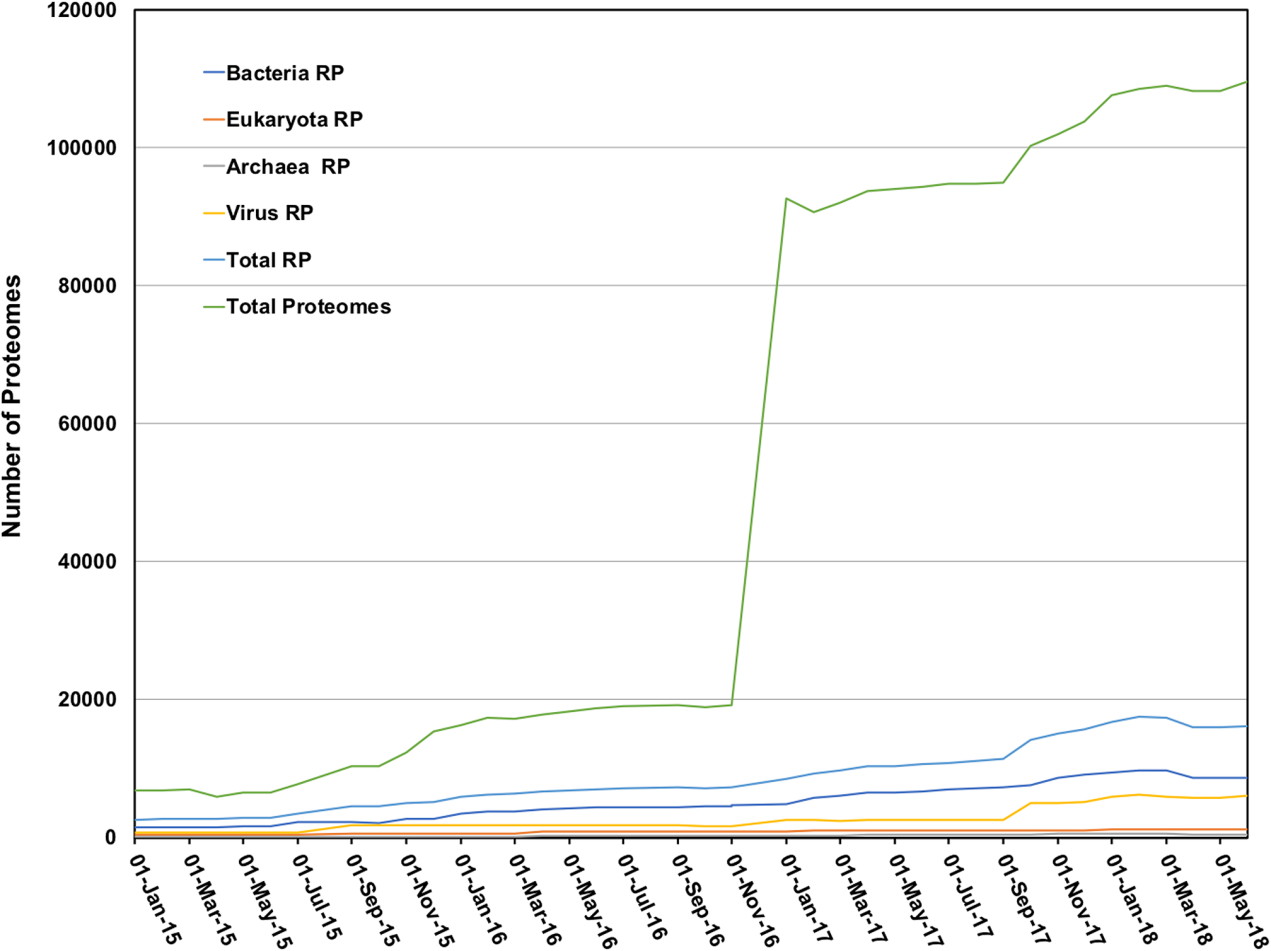
Growth of the total number of Complete Proteomes and Reference Proteomes since 2015.

For the remaining proteomes we provide a Reference Proteome set (∼9% of total proteomes) selected by the research community and supplemented with selected proteomes from a computational clustering (6) to provide the best annotated proteome in their cluster. Recently, we have added virus Reference Proteomes (described below) to this list. The growth of Reference Proteome sets is shown in Figure 2. Programmatic access to the non-redundant proteomes included in UniProtKB is provided via the Proteins API (https://www.ebi.ac.uk/proteins/api/doc/) (7) whilst all proteomes reference, non-reference and redundant in UniProtKB and UniParc are accessible via the Proteome section of the UniProt website (https://www.uniprot.org/proteomes/).

UniProt reference proteomes are used by user communities such as the Quest for Orthologs (QfO) group as the ‘gold standard’ reference proteome dataset for orthologue benchmarking (8). The QfO reference proteomes datasets are a compiled subset of the UniProt reference proteomes, comprising well-annotated model organisms and organisms of interest for biomedical research and phylogeny, with the intention to provide broad coverage of the tree of life whilst maintaining a low number of proteomes for the benchmark. Such datasets have been generated annually from the UniProt Knowledgebase (UniProtKB) database for the past seven years. To this end, a gene-centric pipeline has been developed and enhanced over the past year by UniProt. These complete, non-redundant reference proteomes are publicly available at ftp://ftp.ebi.ac.uk/pub/databases/reference_proteomes/QfO. The datasets are provided either in SeqXML format or as a collection of FASTA files and include genomics coordinates for most proteins. In the last release for QfO in April, the number of species increased from 66 to 78 comprising 1,311,679 non-redundant protein sequences (48 Eukaryotes and 30 bacteria-archaea).

At the time of our previous publication, the number of virus Reference Proteomes in UniProtKB stood at 428—practically an order of magnitude less than the number of known viral species (which stands at 4405 according to the International Committee on Taxonomy of Viruses, or ICTV). To improve Reference Proteome Coverage of viruses in UniProt we have undertaken a concerted effort to curate Complete Proteomes and to use these as input for the computational selection of Reference Proteomes. We compiled and curated complete viral genome (proteome) data from INSDC (which feeds into UniProt) as well as a range of other specialized viral genome resources including ViPr (9), IRD (10), the HIV database (https://www.hiv.lanl.gov/), Papillomavirus Episteme (11) and the HCV database (https://hcv.lanl.gov/) and HBV database (12). This curation effort included the identification and removal of segmented viruses lacking one or more segments, and increased the number of the Complete Proteomes of viral origin in UniProt to 86 951 (UniProt release 2018_07). Computational clustering of this enhanced viral Complete Proteome set (13) produced 5,887 viral Reference Proteomes. Note that redundancy removal procedures are not currently applied to viruses, due to the challenges posed by the high number of variants in the small genomes of these species.

Technological advances have enabled the sequencing of the genetic material from all the microorganisms in a particular environment without the cultivation of any of the community members. Concurrent advances in bioinformatics have enabled the rapid assembly of genomes from metagenomes (MAGs) and a corresponding input of sequence data into the UniProt database with >4800 assembled proteomes in the database (4839, release 2018_07). The existing data input pipeline is currently based on those submissions to the INSDC which fulfil certain threshold criteria but future plans are to move to using the EBI Metagenomics resource, MGnify (14), as the main source of metagenome derived assemblies. We will include only those MAGs that show a high level of completeness and a low level of contamination.

### Expert curation progress

Expert curation of the literature is critically important to the UniProt databases. The information extracted from scientific publications is stored in the UniProtKB/Swiss-Prot section of the UniProt Knowledgebase and describes functional information both in the form of human readable free-text/controlled syntax summaries and via structured vocabularies such as the Gene Ontology (GO) (15) or ChEBI (16). Expert curation is labour intensive, with curators assimilating and evaluating multiple lines of evidence from the text and figures of relevant publications, but this has repeatedly proven to be the most efficient method of extracting all relevant data from a paper. We have previously demonstrated that UniProtKB expert biocuration captures between 35 and 45% of the curatable literature for any given species, rising to 50% of the curatable literature for *Homo sapiens* (17). UniProtKB/Swiss-Prot entries serve as a source of functional data for the development and enhancement of bioinformatics prediction tools, so we prioritize the capture of functional data that cannot currently be correctly predicted by computational methods, such as proteins which predictive protein signatures would describe as being part of an enzyme family but are in fact non-functional due to the loss of specific amino acid residues. An example of this is PLC1 (UniProtKB:Q15111), a member of the phosphoinositide phospholipase C family (InterPro:IPR001192) in which the existence of an asparagine residue in the active site instead of the conserved histidine residue suggests a non-catalytic role for this protein. This has broader applications than the annotation of just one protein as information on protein function is computationally transferred from UniProtKB/Swiss-Prot to sequence-related, but less well studied, proteins in UniProtKB/TrEMBL. Therefore, it is important to ensure we have a broad enough collection of annotated proteins in UniProtKB/Swiss-Prot to add value to new entries as the taxonomic range of fully sequenced proteomes continues to expand.

Once a record has been moved into UniProtKB/Swiss-Prot from UniProtKB/TrEMBL, there is a concerted effort to ensure that it is regularly updated to ensure the current understanding of a protein’s activity reflects that in the scientific literature. This task can be difficult, as our knowledge of protein function continues to evolve and finer grained experimental techniques provide new knowledge that may appear to contradict previous observations. An example of this is provided by the proteins involved in N^6^-methyladenosine (m^6^A) modification which takes place on both coding and non-coding RNAs containing a Pu[G>A]m^6^AC[A/C/U] (Pu = purine) sequence, the nucleotide A3 of which becomes N6-methylated. The modification acts as a key regulator of mRNA stability: methylation is completed upon the release of mRNA into the nucleoplasm and affects various processes, such as mRNA stability, processing, translation efficiency and editing. The enzymes that catalyse this process were believed to have been fully characterized some years ago, but more recent data has changed our understanding of how these molecules work.

m^6^A methylation is performed by a heteromeric WMM N^6^-adenosine-methyltransferase complex, originally thought to be a heterotrimer of METTL3 (UniProtKB:Q86U44), METTL14 (UniProtKB:Q9HCE5) and WTAP (UniProtKB:Q15007), but additional component subunits have recently been identified (18). Initial work indicated that METTL3 and METTL14 form a tight heterodimer in order to perform catalysis on the preferred motif sequence. Both proteins are members of the MT-A70 family and are classified as S-adenosyl-L-methionine-dependent methyltransferases by sequence prediction resources. Methyltransferase activity has been reported for both (19). Subsequent structural studies have now shown that that only one protein, METTL3, constitutes the catalytic subunit (20–22). The other subunit, METTL14, has a degenerate active site that is unable to accommodate donor and acceptor substrates and plays a non-catalytic role in maintaining complex integrity and substrate RNA binding (22). WTAP appears to serve as a regulatory subunit. A newly identified subunit of the WMM methyltransferase complex, VIRMA (UniProtKB:Q69YN4) plays a role in mediating mRNA m^6^A methylation in 3′UTRs and near stop codons and in recruiting the methyltransferase core components.

**Figure 3.**
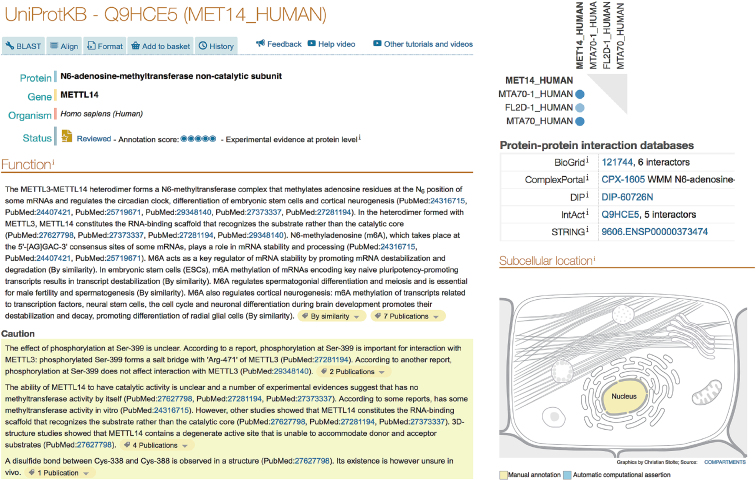
Functional annotation describing human METTL14 (UniProtKB Q9HCE5).

Expert biocuration has been essential in keeping the relevant UniProtKB/Swiss-Prot entries up to date with each step in the pathway to reflect our understanding of the function of these proteins and the molecules they associate with. Every piece of knowledge we capture is associated with evidences (see https://www.uniprot.org/help/evidences for further details) to indicate the source of information and we have added a CAUTION comment to the METTL14 entry to both provide some background and inform users that the initially reported methyltransferase activity is an unsafe observation (Figure 3). The correct annotation on the UniProtKB/Swiss-Prot entry will ensure that misleading data is not computationally added to orthologous proteins in UniProtKB/TrEMBL on the basis of an over-predictive protein signature, again highlighting the importance of expert biocuration. Future plans for the manual curation activities in UniProtKB include the development of mechanisms to identify and highlight contradictory information in existing protein entries in order to improve rigor and reproducibility. This example also illustrates the collaborative nature of the UniProt Consortium in that the molecular interactions involved have also been added to the IMEx Consortium (www.imexconsortium.org) dataset by UniProt curators (23), the protein complexes involved curated into the Complex Portal (https://www.ebi.ac.uk/complexportal/) (24) and both the proteins and complexes have been further annotated using GO (http://www.geneontology.org). New terms were added to the GO to enable this and others were updated. All of these data are subsequently reimported back to enrich the appropriate entries in UniProtKB/Swiss-Prot but are stored and maintained by these domain-specific resources.

### Automatic annotation progress

**Figure 4.**
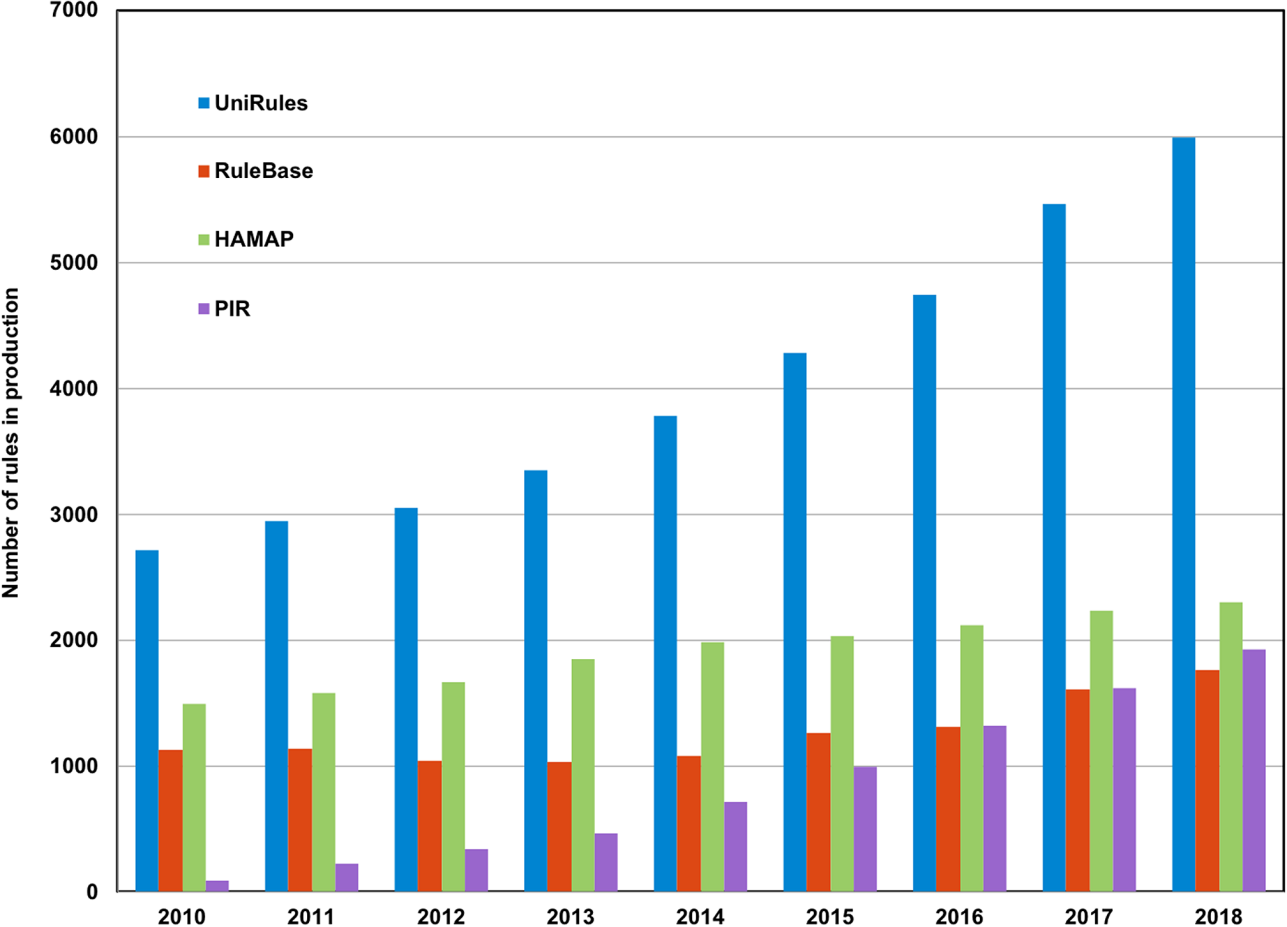
Growth of curated automatic annotation rules within the UniRule system.

UniProt's automatic annotation pipelines enrich the unreviewed records in UniProtKB/TrEMBL with classification and functional annotations. InterPro is used to classify sequences at superfamily, family and subfamily levels and to predict the occurrence of functional domains and important sites. InterPro integrates predictive models of protein function, so-called ‘signatures’, from a number of member databases. In UniProtKB/TrEMBL entries, domains from the InterPro member databases PROSITE, SMART or Pfam are predicted and used to automatically provide domain annotations. All automatic annotations are labelled with their evidence/source. UniProt has developed two complementary rule-based prediction systems, UniRule and the Statistical Automatic Annotation System (SAAS) to automatically annotate UniProtKB/TrEMBL in an efficient and scalable manner with a high degree of accuracy. These prediction systems can annotate protein properties such as protein names, function, catalytic activity, pathway membership and subcellular location, along with sequence-specific information, such as the positions of post-translational modifications and active sites. We continue to increase the number of Rules used for annotation and this has now grown to over 6000 in total as shown in Figure 4.

### Ongoing developments in automatic annotation

We are evaluating new methods for computational annotation of function based on based specifically on their domain architecture (DAAC, or Domain Architecture Alignment and Classification) (22,25) or any combination of sequence properties and features (ARBA, or Association-Rule-Based Annotator).

The Domain Architecture Alignment and Classification system (DAAC, (25)) is based on the assumption that not only the presence of domains is important for the protein to perform its function but also their architecture i.e. their arrangement and order. It performs the prediction in four major steps. Firstly, it generates the different ordered combinations of InterPro domains, these combinations are termed domain architectures (DA). Secondly, it calculates the pairwise similarity between DAs using alignment. The third step is that of training. In this phase, clusters of proteins are created, where each cluster represents a single annotation. A threshold of belonging is determined for each cluster while maximising the classification performance, and then annotations corresponding to low performance clusters are discarded. In the final step, the clusters along with their corresponding thresholds serve as models to annotate other query proteins. The fact that DAAC takes into account the arrangement of domains within proteins allows it to make more accurate predictions for specific cases where proteins can have the same set of domains but with different arrangements and hence could perform different functions. Based on UniProt release 2018_05, DAAC generates 7 095 966 EC predictions. 3 880 775 of them are new predictions (not predicted by UniRule or SAAS). Furthermore, DAAC generates 82 285 267 predictions of GO Terms for 20 827 747 distinct proteins in UniProtKB/TrEMBL. In total, 47 992 323 of the predictions are new (not predicted by UniRule or SAAS).

Association rule mining and selection techniques can be used effectively as computational methods for functional prediction. ARBA (Association-Rule-Based Annotator) is based on these techniques and can be used to enhance the quality of automatically generated annotations as well as annotating proteins with unknown functions. It learns on data from UniProtKB/Swiss-Prot and uses InterPro signatures and organism taxonomy as attributes to predict most of the protein functional annotations including GO terms, metabolic pathways, EC numbers, etc. With respect to certain quality measures, ARBA finds all rules which would define significant relationships between attributes and functional annotations in UniProtKB/Swiss-Prot entries. The set of extracted rules represent the comprehensive knowledge which could explain protein functions. However, these rules comprise redundant information and their high number makes it infeasible to apply them on large sets of data such as UniProtKB/TrEMBL. To address this issue, ARBA puts these rules into a fast competition process called SkyRule based on two concepts, namely dominance and comparability. Rules are then elegantly and considerably reduced in number and aggregated to form concise prediction models that assign functional annotations to UniProtKB entries.

In order to share our knowledge in computational annotation and our rule-based systems, we are working on developing standard formats for rule annotation and tools to apply them. UniFire (the UNIprot Functional annotation Inference Rule Engine) is a standalone tool to apply the UniProt annotation rules on third party data. This engine is based on a Business Rules Management System (BRMS) named Drools and is developed in conjunction with an exchangeable format for UniProt rules and the protein data to be annotated. we termed it URML (Uniprot Rule Markup Language). URML respects the definition of business rules, and is therefore executable. When applied on third party proteins, UniFire will transform the data according to the URML format and execute the UniProt rules on them to generate functional predictions. We would like to work with the scientific community in this development and encourage users to register their interest in the links provide in our blog Inside UniProt (https://insideuniprot.blogspot.com/2018/03/).

A new change in UniRef to provide GO annotation was based on user feedback. The UniRef databases (UniProt Reference Clusters) provide clustered sets of sequences from the UniProt Knowledgebase and selected UniParc records to obtain complete coverage of sequence space at several resolutions while hiding redundant sequences. Our study indicated functional annotations are generally preserved in UniRef clusters due to the intra-cluster homogeneity (26). The GO terms of member protein sequences are highly consistent at UniRef90/50 levels in each of the three GO domains: Molecular Function, Biological Process and Cellular Component. We have started to compute GO annotations last year for UniRef90 and UniRef50 clusters: A GO term is assigned to a cluster when it is found in every UniProtKB member that has GO annotation in this cluster, or when it is a common ancestor of at least one GO term from each such member. We evaluated the GO term prediction by comparing the protein sequences that newly acquired GO annotation with the ones that GO terms were assigned by UniRef in previous releases. The overall rate of prediction precision is higher than 97%, in all three GO domains, indicating the confidence to propagate UniRef GO annotation to member sequences. Thus, UniRef provides an annotation source to our users. They can search against UniRef and then obtain GO annotation from their results.

GO annotations of UniRef can be found in the UniRef XML files from the UniProt ftp site: ftp://ftp.uniprot.org/pub/databases/uniprot/uniref/uniref50/.

For the release 2018_06, 25 300 GO terms were assigned to 33 814 200 UniRef90 clusters and 21 000 GO terms were assigned to 11 235 770 UniRef50 Clusters.

### UniProt bibliography

UniProt compiles additional bibliography from external sources to complement the curated literature set in UniProtKB/Swiss-Prot with additional publications and to add relevant literature to UniProtKB/TrEMBL entries not yet curated. The sources of literature are of two types: biological databases (currently 22 sources, including model organism, structure, post-translational modification, function, disease and interaction databases) where the literature mapped to UniProt entries are added in a collaborative manner, and secondly, text mining (currently PubTator_Tmvar for literature about human variants in disease (27) and pGenN (28) for literature related to plant proteins). Altogether these sources add 902 956 unique papers covering 363 591 entries, with a total of 36 147 401 UniProt accession-PubMed identifier (accession/PMID) pairs (Release 2018_08).

In the entry publication section, publications providing evidence for a specific annotation type are organized across different categories, such as function, interaction and expression, based on the type of data they contain. This categorization facilitates navigation through the protein entry literature by offering users quick access to literature of interest related to a given protein-topic. The classification of the UniProt references is automatically done based on the topics found in the flat file RP lines that are linked to the references, whereas the computationally mapped bibliography has to be categorized in a different way.

As a first approach, we rely on the information provided by the underlying database sources for this categorization (e.g. the literature provided by iPTMnet (29) will fall into PTM/Processing category and literature provided by Intact will be classified as Interaction). However, this approach is not optimal for all literature, thus we are now systematically classifying the articles into the UniProt main categories present in the entry using UPCLASS, a neural network based pipeline to classify publications for UniProtKB protein entries, developed in SIB Text Mining group (https://www.sib.swiss/ruch-patrick/patrick-ruch-sub). UPCLASS has been trained and evaluated with the curated literature and categories in UniProt entries (with precision, recall and F-score of 0.80, 0.62 and 0.70, respectively). The model used for UniProt classification considers the protein names corresponding to the accession-PMID pair, resulting in a more accurate protein-centric classification. Implementation of UPCLASS has enabled the classification of 32 196 740 UniProt accession-PMID pairs that have been previously displayed as unclassified, representing 254 038 protein entries and 768 505 PMIDs (Release 2018_08).

### Outreach and training

The training of our users to make the best use of our data, whether accessed via the web, the API or downloaded from the FTP site, is a key mission of the UniProt Consortium. We have played an active role in the provision of hands-on training workshops across the globe, supporting both early-career researchers and domain specialists such as clinicians and proteomic scientists. Whilst face-to-face training will remain an important route for disseminating very detailed information, it is not always the most efficient method of reaching out to large numbers of users or of penetrating new user groups. We have therefore scheduled a series of webinars, with basic modules on searching protein sequence and function repeated regularly and more specialist units, for example on protein structure, machine learning protein function and linking protein and genome annotation delivered less often. The webinars are recorded, and are subsequently made available online (https://www.ebi.ac.uk/training/online/) where they are supported by related online training materials and YouTube videos (https://www.youtube.com/user/uniprotvideos/). In order to reach new communities, the webinars are widely advertised, making pro-active use of social media forums such as Twitter (@uniprot) and FaceBook (https://www.facebook.com/uniprot.org/) as well as established mailing lists.

### Website developments

During the last year we have added three new visualizations to the UniProt website. Firstly, we have added a method for viewing molecular interactions, secondly a method for viewing the subcellular localization of the proteins and finally we have added a molecular structure viewer. Together these enable users to rapidly understand the molecular context of UniProt entries.

**Figure 5.**
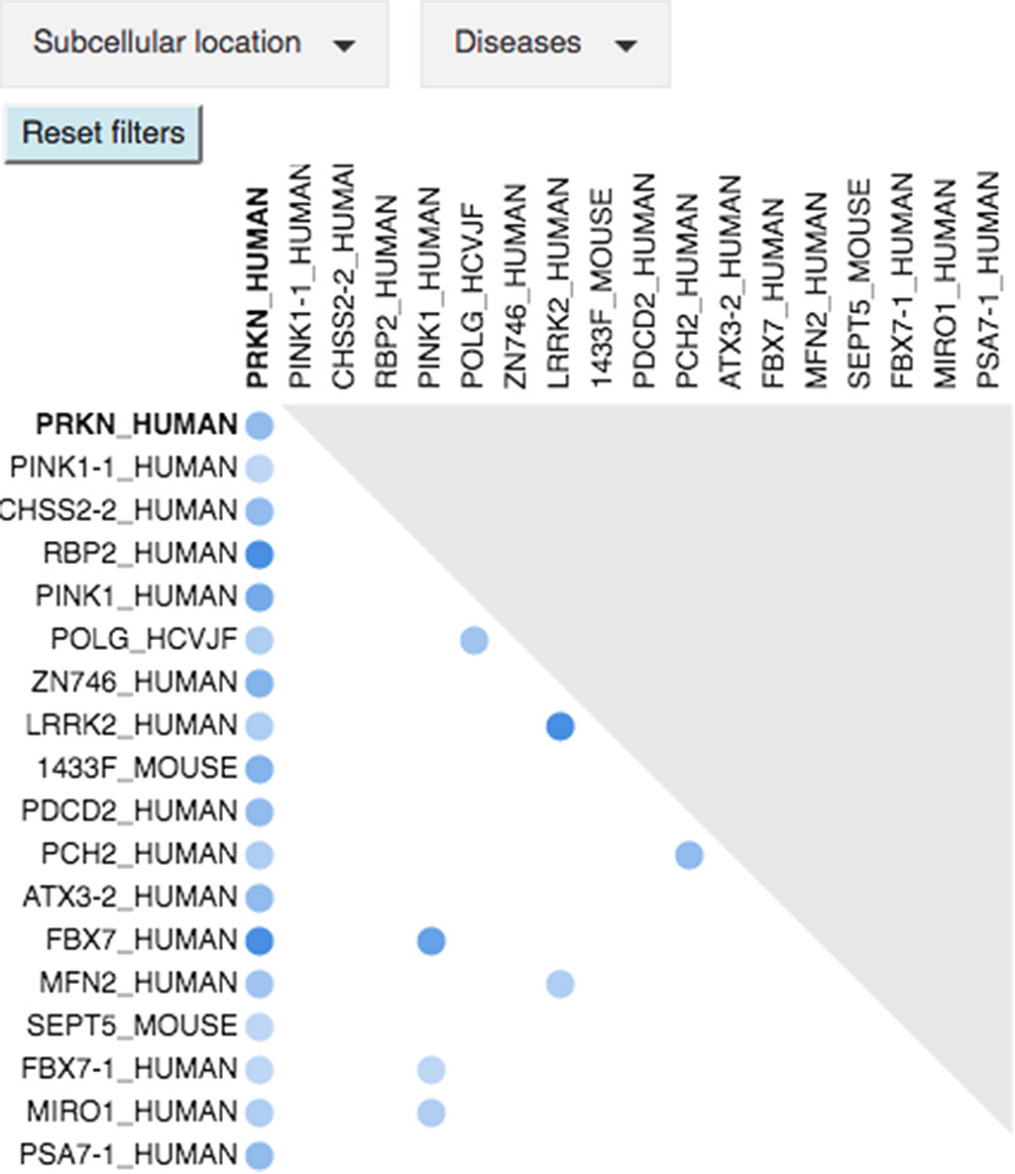
Interaction matrix of the human Parkin protein.

For UniProtKB entries that include an Interaction section, we show details of the protein's binary interactions with other proteins, using a high-quality dataset supplied by the IMEx Consortium. The binary interactions of a protein are now shown as a matrix that shows the interaction partners of your protein and also shows which of those partners interact with each other. For example, the interaction matrix for the human E3 ubiquitin–protein ligase parkin protein (UniProtKB:O60260) is shown in Figure 5.

**Figure 6.**
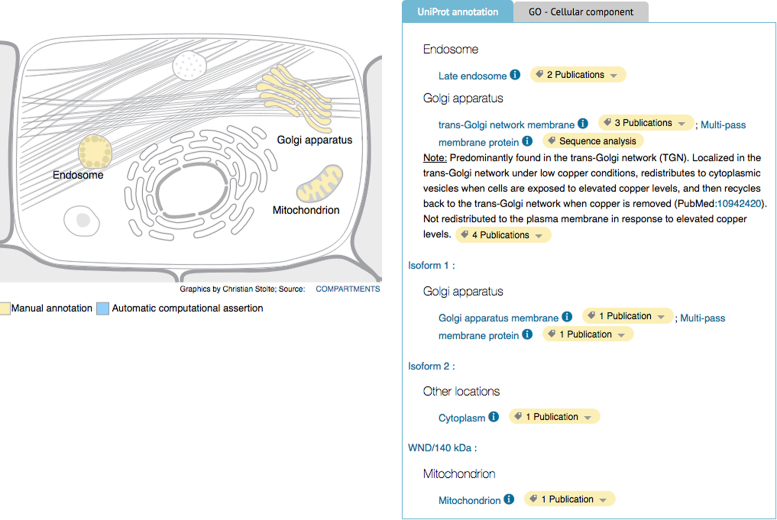
The subcellular localization view of a UniProt entry (UniProtKB P35670).

One of the sections on the protein entry pages is Subcellular Location. This section provides information on the location and the topology of the mature protein in the cell. We now allow users to visually explore the subcellular location in UniProtKB entries. The visualization presents image templates from COMPARTMENTS (https://compartments.jensenlab.org/) (28,30) combined with protein location data from UniProt (expert annotation, rule-based automatic annotation) and imported from GO annotation. Figure 6 below shows the subcellular location view from the Human Copper-transporting ATPase 2 protein (UniProtKB:P35670).

**Figure 7.**
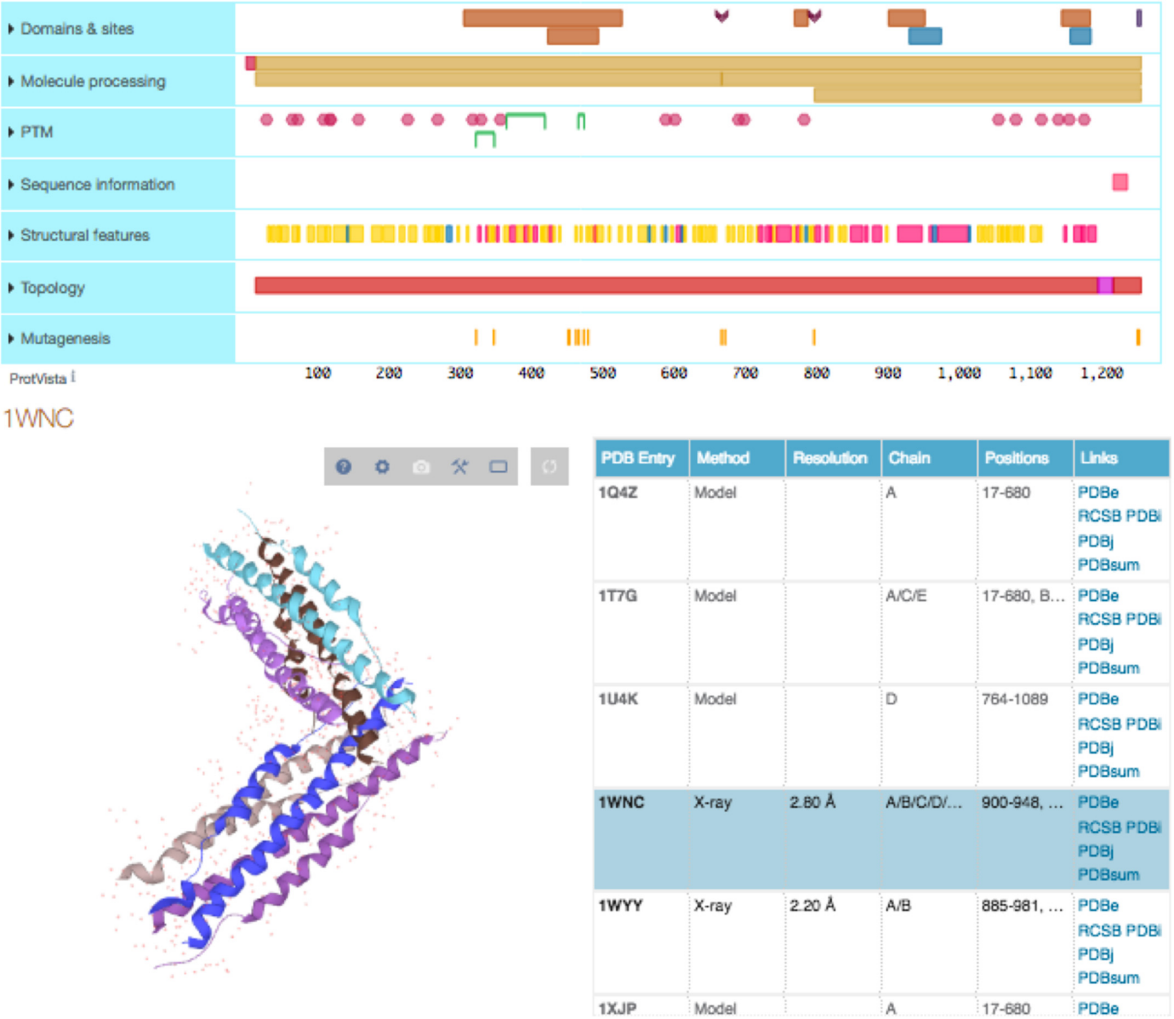
The molecular structure of the Spike protein of the Human SARS coronavirus (PDB ID: 1WNC) structure as shown in the ProtVista protein viewer. The 3D viewer is interactively connected with the sequence level annotations in UniProt e.g. domains, PTMs and mutations. Note that the user can select from any of the structures that map to the protein entry.

Structural information is important in understanding the molecular mechanisms that allow proteins to perform their specific functions. UniProt now provides a protein structure viewer in the ‘Structure’ section of the entry view of the website as well as in the ProtVista protein viewer (see Figure 7). The structures are rendered using the Litemol viewer. This innovation helps users to connect protein information in UniProt with structural data.

## CONCLUSION

UniProt continually develops its processes and procedures to efficiently provide a global collection of protein sequences and annotations. Over the past two years we have seen significant growth in numbers of genomes and proteins sequences. We have continued to organize that data and provide it to our users in a variety of user-friendly ways. We have recently also updated the terms of our license to a CC-BY (4.0) making it easier for UniProt data to be reused by others.

A critical component for UniProt is to connect papers to the relevant entries. In this paper we have described how this is carried out by our expert curators as well as how we supplement these curated papers by connecting papers from other databases and text mining tools. You can help enable curators and text mining tools to connect your scientific papers to UniProt and other molecular biology databases through tagging mentions of proteins with UniProt identifiers. We request that authors use the following format (UniProtKB:P68369) to describe a protein within the text of a paper. This formatting follows the compact identifier representation that has been recently proposed to enable uniform resolution of biomedical resource identifiers (31). Using this format can also provide a simple mechanism to refer to other data resources. Some journals already have specific formatting requirements for such citations to accessions and these should always be given precedence.

We greatly value the feedback and annotation updates from our user community. Please send your feedback and suggestions to the e-mail address help@uniprot.org or via the contact link on the UniProt website.

## References

[1] The UniProt Consortium UniProt: the universal protein knowledgebase. Nucleic Acids Res. 2017; 45:D158–D169. 27899622 10.1093/nar/gkw1099 PMC5210571

[2] Karsch-Mizrachi I., Takagi T., Cochrane G. International Nucleotide Sequence Database Collaboration The international nucleotide sequence database collaboration. Nucleic Acids Res. 2018; 46:D48–D51. 29190397 10.1093/nar/gkx1097 PMC5753279

[3] Zerbino D.R., Achuthan P., Akanni W., Amode M.R., Barrell D., Bhai J., Billis K., Cummins C., Gall A., Girón C.G. Ensembl 2018. Nucleic Acids Res. 2018; 46:D754–D761. 29155950 10.1093/nar/gkx1098 PMC5753206

[4] Giraldo-Calderón G.I., Emrich S.J., MacCallum R.M., Maslen G., Dialynas E., Topalis P., Ho N., Gesing S. VectorBase Consortium VectorBase Consortium Madey G. VectorBase: an updated bioinformatics resource for invertebrate vectors and other organisms related with human diseases. Nucleic Acids Res. 2015; 43:D707–D713. 25510499 10.1093/nar/gku1117 PMC4383932

[5] Howe K.L., Bolt B.J., Shafie M., Kersey P., Berriman M. WormBase ParaSite—a comprehensive resource for helminth genomics. Mol. Biochem. Parasitol. 2017; 215:2–10. 27899279 10.1016/j.molbiopara.2016.11.005 PMC5486357

[6] Chen C., Natale D.A., Finn R.D., Huang H., Zhang J., Wu C.H., Mazumder R. Representative proteomes: a stable, scalable and unbiased proteome set for sequence analysis and functional annotation. PLoS One. 2011; 6:e18910. 21556138 10.1371/journal.pone.0018910 PMC3083393

[7] Nightingale A., Antunes R., Alpi E., Bursteinas B., Gonzales L., Liu W., Luo J., Qi G., Turner E., Martin M. The Proteins API: accessing key integrated protein and genome information. Nucleic Acids Res. 2017; 45:W539–W544. 28383659 10.1093/nar/gkx237 PMC5570157

[8] Altenhoff A.M., Boeckmann B., Capella-Gutierrez S., Dalquen D.A., DeLuca T., Forslund K., Huerta-Cepas J., Linard B., Pereira C., Pryszcz L.P. Standardized benchmarking in the quest for orthologs. Nat. Methods. 2016; 13:425–430. 27043882 10.1038/nmeth.3830 PMC4827703

[9] Pickett B.E., Sadat E.L., Zhang Y., Noronha J.M., Squires R.B., Hunt V., Liu M., Kumar S., Zaremba S., Gu Z. ViPR: an open bioinformatics database and analysis resource for virology research. Nucleic Acids Res. 2012; 40:D593–D598. 22006842 10.1093/nar/gkr859 PMC3245011

[10] Zhang Y., Aevermann B.D., Anderson T.K., Burke D.F., Dauphin G., Gu Z., He S., Kumar S., Larsen C.N., Lee A.J. Influenza research database: An integrated bioinformatics resource for influenza virus research. Nucleic Acids Res. 2017; 45:D466–D474. 27679478 10.1093/nar/gkw857 PMC5210613

[11] Van Doorslaer K., Li Z., Xirasagar S., Maes P., Kaminsky D., Liou D., Sun Q., Kaur R., Huyen Y., McBride A.A. The Papillomavirus Episteme: a major update to the papillomavirus sequence database. Nucleic Acids Res. 2017; 45:D499–D506. 28053164 10.1093/nar/gkw879 PMC5210616

[12] Hayer J., Jadeau F., Deléage G., Kay A., Zoulim F., Combet C. HBVdb: a knowledge database for Hepatitis B Virus. Nucleic Acids Res. 2013; 41:D566–D570. 23125365 10.1093/nar/gks1022 PMC3531116

[13] Chen C., Huang H., Mazumder R., Natale D.A., McGarvey P.B., Zhang J., Polson S.W., Wang Y., Wu C.H., Consortium UniProt Computational clustering for viral reference proteomes. Bioinformatics. 2016; 32:2041–2043. 27153712 10.1093/bioinformatics/btw110 PMC4920120

[14] Mitchell A.L., Scheremetjew M., Denise H., Potter S., Tarkowska A., Qureshi M., Salazar G.A., Pesseat S., Boland M.A., Hunter F.M.I. EBI Metagenomics in 2017: enriching the analysis of microbial communities, from sequence reads to assemblies. Nucleic Acids Res. 2018; 46:D726–D735. 29069476 10.1093/nar/gkx967 PMC5753268

[15] Gene Ontology Consortium The Gene Ontology project in 2008. Nucleic Acids Res. 2008; 36:D440–D444. 17984083 10.1093/nar/gkm883 PMC2238979

[16] Hastings J., Owen G., Dekker A., Ennis M., Kale N., Muthukrishnan V., Turner S., Swainston N., Mendes P., Steinbeck C. ChEBI in 2016: Improved services and an expanding collection of metabolites. Nucleic Acids Res. 2016; 44:D1214–D1219. 26467479 10.1093/nar/gkv1031 PMC4702775

[17] Poux S., Arighi C.N., Magrane M., Bateman A., Wei C.-H., Lu Z., Boutet E., Bye-A-Jee H., Famiglietti M.L., Roechert B. On expert curation and scalability: UniProtKB/Swiss-Prot as a case study. Bioinformatics. 2017; 33:3454–3460. 29036270 10.1093/bioinformatics/btx439 PMC5860168

[18] Yue Y., Liu J., Cui X., Cao J., Luo G., Zhang Z., Cheng T., Gao M., Shu X., Ma H. VIRMA mediates preferential mA mRNA methylation in 3′UTR and near stop codon and associates with alternative polyadenylation. Cell Discov. 2018; 4:10. 29507755 10.1038/s41421-018-0019-0 PMC5826926

[19] Liu J., Yue Y., Han D., Wang X., Fu Y., Zhang L., Jia G., Yu M., Lu Z., Deng X. A METTL3-METTL14 complex mediates mammalian nuclear RNA N6-adenosine methylation. Nat. Chem. Biol. 2014; 10:93–95. 24316715 10.1038/nchembio.1432 PMC3911877

[20] Wang X., Feng J., Xue Y., Guan Z., Zhang D., Liu Z., Gong Z., Wang Q., Huang J., Tang C. Structural basis of N(6)-adenosine methylation by the METTL3-METTL14 complex. Nature. 2016; 534:575–578. 27281194 10.1038/nature18298

[21] Wang P., Doxtader K.A., Nam Y. Structural basis for cooperative function of Mettl3 and Mettl14 Methyltransferases. Mol. Cell. 2016; 63:306–317. 27373337 10.1016/j.molcel.2016.05.041 PMC4958592

[22] Śledź P., Jinek M. Structural insights into the molecular mechanism of the m(6)A writer complex. Elife. 2016; 5:e18434. 27627798 10.7554/eLife.18434 PMC5023411

[23] Orchard S., Kerrien S., Abbani S., Aranda B., Bhate J., Bidwell S., Bridge A., Briganti L., Brinkman F.S.L., Cesareni G. Protein interaction data curation: the International Molecular Exchange (IMEx) consortium. Nat. Methods. 2012; 9:345–350. 22453911 10.1038/nmeth.1931 PMC3703241

[24] Meldal B.H.M., Forner-Martinez O., Costanzo M.C., Dana J., Demeter J., Dumousseau M., Dwight S.S., Gaulton A., Licata L., Melidoni A.N. The complex portal–an encyclopaedia of macromolecular complexes. Nucleic Acids Res. 2015; 43:D479–D484. 25313161 10.1093/nar/gku975 PMC4384031

[25] Doğan T., MacDougall A., Saidi R., Poggioli D., Bateman A., O’Donovan C., Martin M.J. UniProt-DAAC: domain architecture alignment and classification, a new method for automatic functional annotation in UniProtKB. Bioinformatics. 2016; 32:2264–2271. 27153729 10.1093/bioinformatics/btw114 PMC4965628

[26] Suzek B.E., Wang Y., Huang H., McGarvey P.B., Wu C.H., Consortium UniProt UniRef clusters: a comprehensive and scalable alternative for improving sequence similarity searches. Bioinformatics. 2015; 31:926–932. 25398609 10.1093/bioinformatics/btu739 PMC4375400

[27] Wei C.-H., Phan L., Feltz J., Maiti R., Hefferon T., Lu Z. tmVar 2.0: integrating genomic variant information from literature with dbSNP and ClinVar for precision medicine. Bioinformatics. 2018; 34:80–87. 28968638 10.1093/bioinformatics/btx541 PMC5860583

[28] Ding R., Boutet E., Lieberherr D., Schneider M., Tognolli M., Wu C.H., Vijay-Shanker K., Arighi C.N. eGenPub, a text mining system for extending computationally mapped bibliography for UniProt Knowledgebase by capturing centrality. Database. 2017; 2017:doi:10.1093/database/bax081. 10.1093/database/bax081 PMC5691349 29220476

[29] Ross K.E., Arighi C.N., Ren J., Huang H., Wu C.H. Construction of protein phosphorylation networks by data mining, text mining and ontology integration: analysis of the spindle checkpoint. Database. 2013; 2013:bat038. 23749465 10.1093/database/bat038 PMC3675891

[30] Binder J.X., Pletscher-Frankild S., Tsafou K., Stolte C., O’Donoghue S.I., Schneider R., Jensen L.J. COMPARTMENTS: unification and visualization of protein subcellular localization evidence. Database. 2014; 2014:bau012. 24573882 10.1093/database/bau012 PMC3935310

[31] Wimalaratne S.M., Juty N., Kunze J., Janee G., McMurry J.A., Beard N., Jimenez R., Grethe J.S., Hermjakob H., Martone M.E. Uniform resolution of compact identifiers for biomedical data. Sci. Data. 2018; 5:180029. 29737976 10.1038/sdata.2018.29 PMC5944906

